# Main causes and financial impact of whole blood and blood components wastage: a descriptive study, Blood Center of the State Health Department of Rio Grande do Sul, 2018–2022 

**DOI:** 10.1590/S2237-96222026v35e20251152.en

**Published:** 2026-06-12

**Authors:** Pabline da Rosa Tolfo, Roger dos Santos Rosa

**Affiliations:** 1 Universidade Federal do Rio Grande do Sul Porto Alegre RS Brazil Universidade Federal do Rio Grande do Sul, Porto Alegre, RS, Brazil

**Keywords:** Hemotherapy Service, Blood Transfusion, Health Economics and Organizations, Health System Financing, Cross-Sectional Studies, Servicio de Hemoterapia, Transfusión Sanguínea, Economía y Organizaciones para la Atención de la Salud, Financiación de los Sistemas de Salud, Estudios Transversales

## Abstract

**Objective:**

To identify the main causes and the financial impact of whole blood and blood component wastage in a Brazilian blood center between 2018 and 2022.

**Methods:**

Descriptive study using secondary data from the Blood Center of the State Health Department of Rio Grande do Sul. The financial impact was calculated by multiplying the physical data by the reimbursement value for the hemotherapy services under the Brazilian Unified Health System (Sistema Único de Saúde, SUS).

**Results:**

Between 2018 and 2022, the blood center produced 169,575 blood units, with an annual mean (M) of 33,915 (standard deviation [SD] 3,096.8), of which 50,947 (30.0%) were discarded. Fresh frozen plasma was the most discarded blood component (62.8%) and had the lowest utilization rate (28.1%). The main causes of discard were volume outside parameters (7,599 units; 14.9%) and excess stock (7,454 units; 14.6%). Reactive results in serological tests accounted for 7.7% of discards (3,948 units). Among the discarded units, 25,784 (50.6%) were attributed to potentially avoidable causes, resulting in an estimated financial impact of BRL 3.5 million (approximately BRL 702,000 per year).

**Conclusion:**

Despite high production and utilization rates, discards represented substantial quantitative and financial waste.

## Resumen

**Objetivo:**

Identificar las principales causas y el impacto financiero de los descartes de sangre total y de hemocomponentes en un centro de hemoterapia brasileño durante el período 2018-2022.

**Métodos:**

Estudio descriptivo realizado con datos secundarios del Centro de Hemoterapia de la Secretaría de Salud del Estado de Rio Grande do Sul. El impacto financiero se calculó multiplicando los datos físicos por el valor del reembolso del Sistema Único de Salud (Sistema Único de Saúde, SUS) para los servicios de hemoterapia.

**Resultados:**

Entre 2018 y 2022, el centro produjo 169.575 unidades de sangre, con un promedio anual (M) de 33.915 (desviación estándar [SD] de 3.096,8), de las cuales 50.947 (30,0%) fueron descartadas. El plasma fresco congelado fue el hemocomponente más descartado (62,8%) y el que presentó el menor porcentaje de aprovechamiento (28,1%). Las principales causas de descarte fueron el volumen fuera de parámetros (7.599 unidades; 14,9%) y el exceso de stock (7.454 unidades; 14,6%). Los resultados reactivos en pruebas serológicas justificaron el 7,7% de los descartes (3.948 unidades). Entre los descartes, 25.784 unidades (50,6%) se atribuyeron a causas potencialmente evitables, lo que generó un impacto financiero estimado de R$ 3,5 millones (aproximadamente R$ 702 mil anuales).

**Conclusión:**

A pesar de la elevada producción y del alto porcentaje de aprovechamiento, los descartes representaron un desperdicio cuantitativo y financiero significativo.

**Ethical aspects**

This research respected ethical principles, having obtained the following approval data::

Research ethics committee: Escola de Saúde Pública da Secretaria de Saúde do Rio Grande do Sul

Opinion number: 6,581,607

Approval date: 14/12/2023

Certificate of submission for ethical appraisal: 75145323.4.0000.5347

Informed consent form: Dismissed.

## Introduction

Blood transfusions are not risk-free procedures. To minimize hazards and ensure process safety, donation is guided by anonymity and the altruistic, voluntary, and non-remunerated nature of donation. Complementing this strategy, donor selection is rigorous and staged, comprising recruitment, registration, clinical-epidemiological screening, and whole-blood collection (1–3).

From whole blood units, differential centrifugation allows the production of so-called blood components, fractionated into erythrocytes, plasma, and platelets (1). Through physicochemical methods, it is also possible to obtain blood derivatives from plasma, such as immunoglobulins and coagulation factors (4–5).

To maintain transfusion safety, hemotherapy services must adhere to best practices throughout the blood production cycle, in accordance with applicable legislation, and dispose of materials when noncompliance with established requirements is identified to prevent recipient harm (6).

The discard of blood components, as foreseen in current legislation (7), primarily results from rigorous visual inspection aimed at identifying nonconformities such as color and volume alterations, absence of swirling, lipemia in the supernatant, presence of clots or aggregates, or breaches in system integrity (leakage). Unit exclusion also occurs due to reactivity in serological and molecular laboratory tests. Units are further rendered unsuitable due to failures in storage protocols or incidents during transportation.

It is also required that services perform systematic quality control of produced blood components (8), monitoring minimum parameters such as microbiological contamination, degree of hemolysis, and residual red blood cells, which must be conducted in at least 1% of production. 

Given the social and financial costs of a blood unit and the financial impact of unnecessary (potentially avoidable) discards, it is relevant to survey this production and evaluate utilization and discards. The study aims to help reduce waste and increase stock availability, considering the social repercussions and the necessary awareness regarding blood donation.

The objective of this research was to identify the main causes and estimate the financial impact of whole blood and blood component discards at the Blood Center of the State Health Department of Rio Grande do Sul from 2018 to 2022.

## Methods

### Study design

This is a descriptive study based on secondary data collected from health services in Rio Grande do Sul during 2018–2022 (9–11). The temporal frame begins with regulatory changes implemented by the Ministry of Health’s Consolidation Ordinance No. 5, which came into effect at the end of 2017 (12).

### Setting

Data on the production and discard of whole blood and blood components from the Blood Center of the State Health Department of Rio Grande do Sul were analyzed to describe the number of units produced and measure the volume of discarded products, as well as their main causes. The study also sought to identify the utilization of units by health services and estimate the financial impact of avoidable losses. Data collection was carried out in March and April 2024.

### Data sources

The data were collected at the Blood Center of the State Health Department of Rio Grande do Sul following acceptance of the research proposal, through the Institutional Consent Form and approval by the Committee for Ethics in Research on Human Beings of the Federal University of Rio Grande do Sul and the Ethics Committee of the State Health Department of Rio Grande do Sul.

At the blood center, information was obtained from the Management System for Hemotherapy Services (*Sistema de Gerenciamento em Serviços de Hemoterapia*, HEMOVIDA), an information system developed specifically for blood banks, to computerize data from the Brazilian blood cycle (13). Filters and selections were applied to ensure research reproducibility.

### Variables

The variables “units produced,” “units discarded,” and “reason for discard” for total units and each blood component were obtained from the blood center database. All quantities and reasons were specified by type of blood component (red blood cell concentrate, platelet concentrate, cryoprecipitate, fresh frozen plasma, and whole blood) and by year analyzed. The corresponding codes from the Table of Procedures, Medicines, Orthotics, Prosthetics and Special Materials of the Brazilian Unified Health System (*Sistema de Gerenciamento da Tabela de Procedimentos, Medicamentos e OPM do SUS*, SIGTAP) were 03.06.01.001-1, 03.06.02.006-8, 03.06.02.007-6, 03.06.02.008-4, 03.06.02.010-6, 02.12.01.001-8, 02.12.01.005-0, and 02.12.01.006-9.

Discard reasons were classified as potentially avoidable or unavoidable, according to the classification used in a 2019 study (14).

The reimbursement value (in Brazilian reais, BRL) corresponds to the reference amount for reimbursement per blood component for *Brazilian Unified Health System* (*Sistema Único de Saúde*, SUS) hemotherapy services and private institutions, as specified in Annex V of Ordinance (12), in effect during the study period and still valid in 2025.

### Bias

Reasons for whole blood and blood component discards were manually entered into the system by different users, which may lead to varied interpretations of the criteria used, potential inconsistencies in the information, and possible errors in the classification and selection of recorded discard reasons.

### Study size

All discards of whole blood and blood components carried out by the Blood Center of the State Health Department of Rio Grande do Sul, as well as their production, recorded in the HEMOVIDA from 2018 to 2022, were included in the study. Of a total of 50,947 records, 28 (0.05%) were excluded due to incomplete or incorrect information regarding the discards.

### Statistical methods

Among the discarded units, losses classified as potentially avoidable were multiplied by the reimbursement value (12). Values were converted into US dollars (USD), using USD 1=BRL 5.1558 (the average for 2020, the midpoint year of the 2018–2022 period), and adjusted for purchasing power parity (PPP) at the same midpoint year (Int$ 1=BRL 2.26) (15) to facilitate comparisons. The calculated values were rounded to the nearest whole unit. 

Data were collected using filters and selection tools available in the Blood Center’s information system. They were transcribed into Microsoft Excel spreadsheets, and descriptive statistics were computed, including absolute and relative frequencies (where applicable) and summary measures such as the mean and standard deviation. Results were presented in tables, all generated from the study data.

## Results

A total of 169,575 blood units were produced during the 2018–2022 period (Table 1), with an annual mean of 33,915 units (standard deviation [SD] ± 3,096.8 and coefficient of variation [CV] of 9.1%). There was little variation across the years. The highest volume was observed in 2022, accounting for 23% of the total, and the lowest in 2019, at 18.2%. The percentage variation between years was approximately 5%. 

**Table 1:** Absolute frequency (n) and percentage (%) of whole blood units or blood components produced. Blood Center of the State Health Department of Rio Grande do Sul, 2018–2022 (n=169,575)

Blood components or whole blood produced	Years					Total n (%)
	2018	2019	2020	2021	2022	
	n (%)	n (%)	n (%)	n (%)	n (%)	
Red blood cell concentrate	18,494 (56.6)	16,740 (53.9)	15,622 (48.4)	16,394 (47.2)	17,784 (45.7)	85,034 (50.1)
Platelet concentrate	4,323 (13.2)	5,584 (18.0)	7,211 (22.4)	8,014 (23.1)	9,547 (24.5)	34,679 (20.5)
Cryoprecipitate	396 (1.2)	499 (1.6)	270 (0.8)	479 (1.4)	462 (1.2)	2,106 (1.3)
Fresh frozen plasma	8,728 (26.7)	7,565 (24.4)	8,597 (26.7)	9,125 (26.3)	10,496 (27.0)	44,511 (26.2)
Whole blood	741 (2.3)	649 (2.1)	535 (1.7)	681 (2.0)	639 (1.6)	3,245 (1.9)
Total	32,682 (100)	31,037 (100)	32,235 (100)	34,693 (100)	38,928 (100)	169,575 (100)

There was also little annual percentage variation regarding the production of blood components. Red blood cell concentrate accounted for the highest share in 2018 (56.6%) and the lowest in 2022 (45.7%) (Table 1). Platelet concentrate and fresh frozen plasma stood out in 2022, exceeding by 11.3 and 2.6 percentage points, respectively, the percentages in the years when they were produced at their lowest levels. Cryoprecipitate was the least produced component, varying from 0.8% of the total in 2020 to 1.6% in 2019.

The discard of blood components over the five years reached 50,947 units (30.0% of the 169,575 blood units produced) (Table 2). The lowest amount of discard occurred in 2019 (8,542 units, 16.8%) and the highest in 2022 (11,818 units, 23.2%). The most frequently discarded blood component was fresh frozen plasma, accounting for 62.8% of the total discarded and having the lowest utilization rate (28.1%). Cryoprecipitate had the lowest discard rate, at 0.5% of the total.

**Table 2:** Absolute frequency (n) and percentage (%) of discarded units of whole blood or blood components. Blood Center of the State Health Department of Rio Grande do Sul, 2018–2022 (n=50,947)

Discarded whole blood or blood components	Years					Total n (%)
	2018 n (%)	2019 n (%)	2020 n (%)	2021 n (%)	2022 n (%)	
Red blood cell concentrate	1,194 (11.2)	1,256 (14.7)	1,067 (11.0)	962 (9.4)	1,002 (8.5)	5,481 (10.8)
Platelet concentrate	1,513 (14.2)	1,418 (16.6)	2,036 (21.1)	2,358 (22.9)	2,658 (22.5)	9,983 (19.6)
Cryoprecipitate	32 (0.4)	93 (1.1)	52 (0.6)	30 (0.3)	51 (0.4)	258 (0.5)
Fresh frozen plasma	7,178 (67.4)	5,128 (60.0)	5,981 (61.9)	6,259 (60.9)	7,472 (63.2)	32,018 (62.8)
Whole blood	729 (6.8)	647 (7.6)	523 (5.4)	673 (6.5)	635 (5.4)	3,207 (6.3)
Total	10,646 (100)	8,542 (100)	9,659 (100)	10,282 (100)	11,818 (100)	50.947 (100)

Discard reasons were highly diverse. The 16 main causes totaled 76.8% of discarded units (Table 3), with emphasis on volume outside established parameters (7,599 units, 14.9%) and excess stock (7,454 units, 14.6%). These variables were classified into potentially avoidable (11 categories) and unavoidable (five categories) using the methodology from a previous 2019 study (14). The first group totaled 25,784 units (50.6%), with volume discrepancy being the most significant among them.

**Table 3:** Main causes of discarded units of whole blood or blood components. Blood Center of the State Health Department of Rio Grande do Sul, 2018–2022 (n=50,947)

Type	Causes of discard	Blood component or whole blood					Total
		Red blood cell concentrate (n)	Platelet concentrate (n)	Cryoprecipitate (n)	Fresh frozen plasma (n)	Whole blood (n)	n (%)
Avoidable	Volume outside established parameters	5	12	3	7,568	11	7,599 (14.9)
	Expired	732	4,327	24	48	-	5,131 (10.1)
	Red blood cell contamination	-	1,960	-	2,790	-	4,750 (9.3)
	Reactive serology	2,652	809	-	478	9	3,948 (7.7)
	Fractionation incident	25	376	-	935	13	1,349 (2.6)
	Lipemia	-	159	-	734	43	936 (1.8)
	Presence of lumps	-	882	-	-	-	882 (1.7)
	Donor with risky behavior	19	-	-	-	559	578 (1.1)
	Open system	88	38	28	277	66	497 (1.0)
	Absence of swirling	-	101	-	-	-	101 (0.2)
	Inadequate storage	3	3	-	7	-	13 (0.0)
Non-avoidable	Excess stock	1	9	-	7,444	-	7,454 (14.6)
	Abnormal coloration	-	228	-	8,070	-	8,298 (16.3)
	Used for quality control	950	703	202	743	-	2,598 (5.1)
	Insufficient whole blood volume	-	-	-	-	1,995	1,995 (3.9)
	Self-exclusion	217	76	-	72	256	621 (1.2)
	Subtotal	4,687	9,671	254	21,598	2,941	39,151 (76.8)
	Other causes	794	312	4	10,420	266	11,796 (23.2)
	Total	5,481	9,983	258	32,018	3,207	50,947 (100)

To obtain results for the blood components used by the Blood Center of the State Health Department of Rio Grande do Sul during the period (Table 4), values were calculated as production numbers minus discarded units. The components with the highest utilization were red blood cell concentrate, which reached its best utilization rate in 2022 (94.4%), and cryoprecipitate, which reached 93.7% in 2021. Fresh frozen plasma had the lowest yields, ranging from 17.8% in 2018 to 32.2% in 2019. 

**Table 4:** Absolute frequency (n) and percentage (%) of utilization by blood component. Blood Center of the State Health Department of Rio Grande do Sul, 2018–2022 (n=118,590)

Blood component	Years				
	2018	2019	2020	2021	2022
	n (%)	n (%)	n (%)	n (%)	n (%)
Red blood cell concentrate	17,300 (93.5)	15,484 (92.5)	14,555 (93.2)	15,432 (94.1)	16,782 (94.4)
Platelet concentrate	2,810 (65.0)	4,166 (74.6)	5,175 (71.8)	5,656 (70.6)	6,889 (72.2)
Cryoprecipitate	364 (91.9)	406 (81.4)	218 (80.7)	449 (93.7)	411 (89.0)
Fresh frozen plasma	1,550 (17.8)	2,437 (32.2)	2,616 (30.4)	2,866 (31.4)	3,024 (28.8)

The financial impact generated by potentially avoidable discards totaled BRL 3,508,555, equivalent to USD 680,506 or Int$ 1,552,458 (Table 5). Annually, this represented BRL 701,711. It was observed that the blood component generating the highest expense was fresh frozen plasma (45.7%) and the lowest was cryoprecipitate (0.2%).

**Table 5:** Financial impact of potentially avoidable discards. Blood Center of the State Health Department of Rio Grande do Sul, 2018–2022 (n=25,784)

Blood component or whole blood	Reference value (BRL)	Discarded quantity	Total		
			BRL	USD	Int$
Red blood cell concentrate	150	3,524	528,600	102,525	233,894
Platelet concentrate	135	8,667	1,170,045	226,938	517,719
Cryoprecipitate	100	55	5,500	1,067	2,434
Fresh frozen plasma	125	12,837	1,604,625	311,227	710,011
Whole blood	285	701	199,785	38,750	88,400
Total		25,784	3,508,555	680,506	1,552,458

## Discussion

In 2022, Rio Grande do Sul recorded a total production of 103,000 blood units (16), of which 49,321 originated from the state capital, Porto Alegre. In the same year, the Blood Center of the State Health Department of Rio Grande do Sul was responsible for 37.8% of the state’s production and 78.9% of the municipality’s production. 

Also in 2022, based on the selection of coordinating blood centers, the federation units with the highest blood unit production were the states of Rio de Janeiro (n=194,240) and Paraná (n=447,769) (16). The blood center of Rio Grande do Sul presented considerably lower volumes.

Studies show that conducting campaigns, distributing informational booklets on the blood donation process, sending letters and emails, and making phone calls increase donor recruitment through awareness-raising strategies (17–19).

When the years were compared, a decline in donations was observed only in 2019 compared with the previous year. In 2020, although donations increased compared with the immediately preceding year, production remained below 2018 levels. Data indicate that, during the years in question, blood donation records declined by 10% due to the COVID-19 pandemic (20). Another study reported a 38% decline during the same period (21). At the Blood Center of the State Health Department of Rio Grande do Sul, the reduction was not as marked as the figures reported in those studies. 

Regarding blood components, the component profile required for coherent stock management to meet the demand of transfusion agencies consists of 43% red blood cell concentrate and 32% platelet concentrate (22). The blood center’s production approached the expected proportion for red blood cell concentrate, as the five-year average was 50.1%. However, it was well below the expected platelet concentration, which averaged 20.5%. The cited study did not report profiles for the blood components cryoprecipitate and fresh frozen plasma. 

The most produced blood component at the Blood Center of the State Health Department of Rio Grande do Sul in all years was red blood cell concentrate. This finding is consistent with the cited literature, as it is the blood component with the highest distribution. High stock levels are required to maintain a viable supply.

Based on the evaluation of the annual hemotherapy production reports issued by the Brazilian Health Regulatory Agency (*Agência Nacional de Vigilância Sanitária*, Anvisa) (23–25), it was observed that the Brazilian averages and those of the Southern region were very similar over the five years evaluated (2018–2022), as were the values presented by the blood center under study. These figures were also higher than all national percentages when the Southern region as a whole was considered, and they overlapped in the use of platelet and red blood cell concentrates.

Among the blood components analyzed, fresh frozen plasma had the lowest utilization rate, paradoxically due to its high production volume. Because it can be stored for long periods (12 or 24 months), it has a high discard rate due to excess stock and limited storage space. One alternative to reduce this discard is to refer the production of blood derivatives, which can be performed by qualified hemotherapy services (22).

In 2023, the Blood Center of the State Health Department of Rio Grande do Sul began supplying excess plasma to the Brazilian Company of Blood Derivatives and Biotechnology (*Empresa Brasileira de Hemoderivados e Biotecnologia*, Hemobrás), a public company of the Ministry of Health responsible for the production of blood-derived medicines in Brazil and their supply to SUS patients. Plasma thus achieved better utilization of blood components within the public health system, without any commercialization (26).

Most national blood centers do not use whole blood, as they believe that transfusing specific blood components is more effective (27). However, it has been suggested that massive transfusion has proven effective, as it provides a more complete replacement of blood components when implemented in practice with appropriate protocols and trained professionals (28).

In 2018, a national report presenting aggregate data reported that 7,938,696 blood component units were produced, of which 33.26% were discarded (23). When analyzing the same year, the Blood Center of the State Health Department of Rio Grande do Sul recorded 32.6% of discarded blood units, close to the national average. This performance was better than that observed during the previous five-year period, 2013 to 2017, which ranged from 31% to 38% in the number of discarded units.

After categorizing discard reasons, of the 11 classified as potentially avoidable, seven (inadequate storage; absence of swirling; red blood cell contamination; incidents during component separation; presence of aggregates; open system; volume outside parameters) could be attributed to weaknesses in the processes of component separation (centrifugation), temperature control, and storage (2). These factors could be minimized through continuing education to improve the qualifications of professionals who perform blood component separation (29).

Classifying the volume outside established parameters as an avoidable cause is controversial, as it may result from incorrect centrifugation and may also depend on sample-specific factors. Researchers have classified it as avoidable, noting that the latter case can be minimized by donor hydration, adequate nutrition, and rest prior to donation (14). 

The situation is identical with respect to lipemia, characterized by a milky appearance of the sample, which can be reduced by ensuring adequate donor nutrition before blood collection (14). Although lipemia does not directly affect transfused blood components (30), discarding such samples is still recommended, as samples with excessive lipemia may interfere with serological laboratory testing.

One study described the main reasons for donor ineligibility at a Brazilian blood center and found that the primary cause was disease transmission risk (36.6%) (13). Among other causes, discards due to donors with risky behavior and reactive serology fell within these categories, which could have been mitigated by disseminating information about prior testing and donor awareness (5).

The Blood Center of the State Health Department of Rio Grande do Sul has a high production volume; therefore, it must comply with legislation requiring that 1% of its production undergo quality-control testing (8). In this study, discards from quality control testing exceeded the required threshold (5.1%), reflecting a strong commitment to maintaining quality standards; however, this led to a significant number of blood units not being used for transfusion.

Regarding the discard of expired samples, which represents the loss of blood components due to expiration, this is typically high, particularly for platelet concentrates, which have a short therapeutic shelf life (five days) and a high production volume (15). It is noteworthy that services need to balance stock levels with actual demand, thereby equalizing periods of lower turnover throughout the year (22).

When comparing the results of this study with others (15,22), similar values and discard reasons were observed. The highest discard rates were for plasma components, ranging from 60% to 80%, due to overproduction, followed by cryoprecipitate, with 18% to 31% discarded because of short shelf life (23), and finally red blood cell concentrate, with 6% to 12% discarded.

The financial impact on the blood center is estimated based on the time and materials required to collect and test the discarded units (15). These blood components are not supplied, and the reimbursement that would have been provided under current regulations (12) is not carried out.

Another important factor to consider is waste generation, which, in addition to being harmful to the environment and public health (14), also imposes costs on the blood center, as biological waste disposal requires specialized companies (27).

According to this research, between 2018 and 2022, the amount the Blood Center of the State Health Department of Rio Grande do Sul did not receive due to potentially avoidable discards was approximately BRL 3.5 million, or approximately BRL 702,000 per year. The previously mentioned costs, representing financial resource waste, were excluded from this calculation.

Despite the high production and utilization rates at the Blood Center of the State Health Department of Rio Grande do Sul, discards resulted in significant financial losses. Educational measures, such as informational campaigns and professional training, are recommended to reduce losses and improve the availability of blood components to the population. It should be emphasized that avoidable discards also affect stock levels, reducing the number of units available to health services and ultimately impacting the population that could benefit from donations. 

## Data Availability

The database and references used in this research analysis are available at https://osf.io/w58hq.
