# Supplementary material for: Main causes and financial impact of whole blood and blood components wastage: a descriptive study, Blood Center of the State Health Department of Rio Grande do Sul, 2018–2022
Source: Epidemiol Serv Saude. 2026 Jun 12;35:e20251152. doi: 10.1590/S2237-96222026v35e20251152.en (PMC13686377; doi:10.1590/S2237-96222026v35e20251152.en)
Supplement: Translation

# Translation: Principais causas e impacto financeiro de descartes de sangue total e de hemocomponentes: estudo descritivo, Hemocentro da Secretaria Estadual de Saúde do Rio Grande do Sul, 2018-2022

**DOI:** 10.1590/S2237-96222026v35e20251152.pt

**Authors:** Pabline da Rosa Tolfo, Roger dos Santos Rosa

**Affiliations:** 1 Universidade Federal do Rio Grande do Sul Porto Alegre RS Brasil Universidade Federal do Rio Grande do Sul, Porto Alegre, RS, Brasil

**Keywords:** Serviço de Hemoterapia, Transfusão de Sangue, Economia e Organizações de Saúde, Financiamento dos Sistemas de Saúde, Estudos Transversais

## Abstract

**Objetivo:**

Identificar as principais causas e o impacto financeiro de descartes de sangue total e de hemocomponentes em um hemocentro brasileiro no período 2018-2022.

**Métodos:**

Estudo descritivo, realizado com dados secundários do Hemocentro da Secretaria Estadual de Saúde do Rio Grande do Sul. O impacto financeiro foi calculado a partir da multiplicação dos dados físicos pelo valor de ressarcimento do Sistema Único de Saúde aos serviços de hemoterapia.

**Resultados:**

Entre 2018 e 2022, o hemocentro produziu 169.575 bolsas de sangue, com média anual (M) de 33.915 (desvio-padrão [DP] 3.096,8), das quais 50.947 (30,0%) foram descartadas. O plasma fresco congelado foi o hemocomponente mais descartado (62,8%) e o que apresentou o menor percentual de aproveitamento (28,1%). As principais causas de descarte foram volume fora dos parâmetros (7.599 unidades; 14,9%) e estoque excedente (7.454; 14,6%).

**Resultados:**

reagentes em testes sorológicos justificaram 7,7% dos descartes (3.948 unidades). Entre os descartes, 25.784 bolsas (50,6%) foram atribuídas a causas potencialmente evitáveis, o que gerou um impacto financeiro estimado de R$ 3,5 milhões (aproximadamente R$ 702 mil anuais).

**Conclusão:**

Apesar da elevada produção e do alto percentual de aproveitamento, os descartes representaram desperdícios quantitativos e financeiros expressivos.

**Aspectos éticos**

Esta pesquisa respeitou os princípios éticos, obtendo os seguintes dados de aprovação:

Comitê de ética em pesquisa: Escola de Saúde Pública da Secretaria de Saúde do Rio Grande do Sul

Número do parecer: 6.581.607

Data de aprovação: 14/12/2023

Certificado de apresentação de apreciação ética: 75145323.4.0000.5347

Registro do consentimento livre e esclarecido: Dispensado.

## Introdução

As transfusões de sangue não são procedimentos isentos de riscos. Para minimizar perigos e garantir a segurança do processo, a doação é pautada pelo anonimato e pelo caráter altruísta, voluntário e não remunerado. Complementar a essa estratégia, a seleção de doadores é rigorosa e dividida em etapas, que incluem a captação, o cadastro, a triagem clínico-epidemiológica e a coleta de sangue total (1-3).

Das bolsas de sangue total, por meio de centrifugação diferencial, são obtidos os chamados hemocomponentes, fracionados em componentes eritrocitários, plasmáticos e plaquetários (1). Por métodos físico-químicos, é possível obter hemoderivados, oriundos do plasma, como imunoglobulinas e fatores de coagulação (4-5).

Com o objetivo de manter a segurança transfusional, o serviço de hemoterapia deve cumprir as boas práticas aplicadas ao ciclo produtivo do sangue, conforme legislação, com descarte do material em caso de não conformidades dos requisitos estabelecidos, para evitar danos ao receptor (6).

O descarte de hemocomponentes, conforme previsto na legislação vigente (7), decorre primordialmente de inspeção visual rigorosa, que visa identificar inconformidades, como alterações de cor e de volume, ausência de swirling, lipemia do sobrenadante, presença de coágulos e grumos ou falhas na integridade do sistema (vazamentos). A exclusão de unidades também ocorre devido à reatividade em exames laboratoriais sorológicos e moleculares. Inviabilizam-se ainda bolsas comprometidas por falhas nos protocolos de armazenamento ou por intercorrências durante o transporte.

Também é exigido que os serviços realizem controle de qualidade sistemático dos hemocomponentes produzidos (8), com observação de alguns parâmetros mínimos como contaminação microbiológica, grau de hemólise e hemácias residuais, que deve ser realizado em, pelo menos, 1% da produção. 

Tendo-se em vista o custo social e monetário de uma bolsa de sangue e que seu descarte desnecessário (potencialmente evitáveis) causa impacto financeiro, torna-se relevante realizar um levantamento dessa produção e avaliar o aproveitamento e os descartes. Busca-se auxiliar na diminuição do desperdício e no aumento de estoque, com a consideração da repercussão social e da conscientização necessária sobre a doação de sangue.

O objetivo desta pesquisa foi identificar as principais causas e estimar o impacto financeiro de descartes de sangue total e de hemocomponentes no Hemocentro da Secretaria Estadual de Saúde do Rio Grande do Sul no período de 2018 a 2022.

## Métodos

### Desenho do estudo

Trata-se de um estudo descritivo realizado por meio da coleta de dados secundários disponíveis dos serviços de saúde do Rio Grande do Sul no quinquênio de 2018 a 2022 (9-11). O início do marco temporal considera que foram realizadas mudanças na normatização pela Portaria de Consolidação nº 5 do Ministério da Saúde, que entrou em vigor no final de 2017 (12).

### Contexto

Foram analisados os dados de produção e de descarte de sangue total e de hemocomponentes do Hemocentro da Secretaria Estadual de Saúde do Rio Grande do Sul, para descrever o quantitativo de unidades produzidas e mensurar o volume de produtos descartados, bem como seus principais motivos. Também se buscou identificar o aproveitamento das bolsas pelos serviços de saúde e estimar o impacto financeiro decorrente de perdas evitáveis. A coleta foi realizada em março e em abril de 2024.

### Fonte de dados

A coleta dos dados ocorreu no Hemocentro da Secretaria Estadual de Saúde do Rio Grande do Sul, após a aceitação da proposta, por meio do Termo de Anuência Institucional e a aprovação pelo Comitê de Ética em Pesquisa com Seres Humanos da Universidade Federal do Rio Grande do Sul e pelo Comitê de Ética da Secretaria de Saúde do Rio Grande do Sul.

No hemocentro, as informações foram obtidas do HEMOVIDA, sistema de informação desenvolvido especificamente para bancos de sangue, com o objetivo de informatizar os dados do ciclo do sangue do Brasil (13). Filtros e seleções foram utilizados com o fim de garantir a reprodutibilidade da pesquisa.

### Variáveis

As variáveis quantidade produzida, quantidade descartada e motivo de descarte do total de bolsas e de cada hemocomponente foram obtidos do banco de dados do hemocentro. Todas as quantidades e os motivos foram discriminados por tipo de hemocomponente (concentrado de hemácias, concentrado de plaquetas, crioprecipitado, plasma fresco congelado e sangue total) e ano analisado. Os códigos correspondentes da tabela SIGTAP do Sistema Único de Saúde (SUS) foram 03.06.01.001-1, 03.06.02.006-8, 03.06.02.007-6, 03.06.02.008-4, 03.06.02.010-6, 02.12.01.001-8, 02.12.01.005-0 e 02.12.01.006-9.

Os motivos de descarte foram classificados como potencialmente evitáveis e não evitáveis, conforme divisão utilizada em estudo prévio, de 2019 (14).

O valor (em reais, R$) do ressarcimento corresponde ao valor de referência para o ressarcimento por hemocomponente entre os serviços de hemoterapia do Sistema Único de Saúde e de instituições privadas, conforme o anexo V de Portaria (12) vigente no período do estudo e ainda em 2025.

### Viés

Os motivos de descartes de sangue total e de hemocomponentes foram inseridos manualmente no sistema por diferentes usuários, o que pode gerar interpretações distintas dos critérios adotados e possibilidade de inconsistências nas informações, bem como eventuais equívocos na classificação e na seleção dos motivos registrados.

### Tamanho do estudo

Foram abrangidos todos os descartes de sangue e de hemocomponentes realizados pelo Hemocentro da Secretaria Estadual de Saúde do Rio Grande do Sul, bem como sua produção, registrados no sistema HEMOVIDA de 2018 a 2022. De um total de 50.947 registros, foram excluídos 28 (0,05%) por informações incompletas ou equivocadas de dados relativos ao descarte.

### Métodos estatísticos

Dentre o quantitativo descartado, as perdas classificadas como potencialmente evitáveis foram multiplicadas pelo valor de ressarcimento (12). Os valores foram convertidos em dólares norte-americanos (US$), considerando-se 1 US$ = R$ 5,1558 (valor médio de 2020, ano intermediário do período 2018-2022), e colocados em paridade de poder de compra (PPC) do mesmo ano intermediário da série (1 Int$ = R$ 2,26) (15), para facilitar comparações. Os valores calculados foram arredondados ao final para a unidade mais próxima. 

Os dados foram coletados por meio da utilização de filtros e de seleções disponíveis no sistema de informação do Hemocentro. Eles foram transcritos para planilhas no software Microsoft Office Excel e estatística descritiva foi utilizada por meio do cálculo de frequências absolutas e percentuais (quando pertinente) e medidas-resumo como média e desvio-padrão. Os resultados foram apresentados na forma de tabelas, todas geradas utilizando como fonte os dados da pesquisa.

## Resultados

Foram produzidas 169.575 bolsas de sangue no período 2018 a 2022 (Tabela 1), uma média anual de 33.915 bolsas (desvio-padrão, DP, de ± 3.096,8 e coeficiente de variação, CV, de 9,1%). Houve pouca variação entre os anos. O maior volume foi observado em 2022, que representou 23% do total, e o menor em 2019, com 18,2%. A variação percentual entre os anos situou-se próxima a 5%. 

**Tabela 1:** Frequência absoluta (n) e percentual (%) de bolsas de sangue total ou de hemocomponentes produzidos. Hemocentro da Secretaria Estadual de Saúde do Rio Grande do Sul, 2018-2022 (n=169.575)

Hemocomponente ou sangue total produzido	Anos					Total n (%)
	2018	2019	2020	2021	2022	
	n (%)	n (%)	n (%)	n (%)	n (%)	
Concentrado de hemácias	18.494 (56,6)	16.740 (53,9)	15.622 (48,4)	16.394 (47,2)	17.784 (45,7)	85.034 (50,1)
Concentrado de plaquetas	4.323(13,2)	5.584 (18,0)	7.211 (22,4)	8.014 (23,1)	9.547 (24,5)	34.679 (20,5)
Crioprecipitado	396 (1,2)	499 (1,6)	270 (0,8)	479 (1,4)	462 (1,2)	2.106 (1,3)
Plasma fresco congelado	8.728 (26,7)	7.565 (24,4)	8.597 (26,7)	9.125 (26,3)	10.496 (27,0)	44.511 (26,2)
Sangue total	741 (2,3)	649 (2,1)	535 (1,7)	681 (2,0)	639 (1,6)	3.245 (1,9)
Total	32.682 (100)	31.037 (100)	32.235 (100)	34.693 (100)	38.928 (100)	169.575 (100)

Também houve pouca variação percentual por ano em relação à produção de hemocomponentes. O concentrado de hemácias destacou-se em todos os anos, com o máximo de 56,6% do total de 2018 e o mínimo de 45,7% em 2022 (Tabela 1). O concentrado de plaquetas e o plasma fresco congelado destacaram-se em 2022, superando em 11,3% e em 2,6% pontos percentuais, respectivamente, o percentual do ano em que foram menos produzidos. O crioprecipitado foi o componente menos fabricado, com variação entre 0,8% do total em 2020 e 1,6% em 2019.

 O descarte dos componentes sanguíneos, somados os cinco anos, atingiu 50.947 (30,0% das 169.575 bolsas produzidas) (Tabela 2). A menor quantidade de descarte ocorreu em 2019 (8.542 bolsas, 16,8%) e a maior em 2022 (11.818, 23,2%). O hemocomponente mais desprezado foi o plasma fresco congelado, o que representou 62,8% do total descartado, e que teve o menor aproveitamento (28,1%). O crioprecipitado foi o componente que apresentou menos descarte, 0,5% do total.

**Tabela 2:** Frequência absoluta (n) e percentual (%) de bolsas de sangue total ou de hemocomponentes descartados. Hemocentro da Secretaria Estadual de Saúde do Rio Grande do Sul, 2018-2022 (n=50.947)

Hemocomponente ou sangue total descartado	Anos					Total n (%)
	2018 n (%)	2019 n (%)	2020 n (%)	2021 n (%)	2022 n (%)	
Concentrado de hemácias	1.194 (11,2)	1.256 (14,7)	1.067 (11,0)	962 (9,4)	1.002 (8,5)	5.481 (10,8)
Concentrado de plaquetas	1.513 (14,2)	1.418 (16,6)	2.036 (21,1)	2.358 (22,9)	2.658 (22,5)	9.983 (19,6)
Crioprecipitado	32 (0,4)	93 (1,1)	52 (0,6)	30 (0,3)	51 (0,4)	258 (0,5)
Plasma fresco congelado	7.178 (67,4)	5.128 (60,0)	5.981 (61,9)	6.259 (60,9)	7.472 (63,2)	32.018 (62,8)
Sangue total	729 (6,8)	647 (7,6)	523 (5,4)	673 (6,5)	635 (5,4)	3.207 (6,3)
Total	10.646 (100)	8.542 (100)	9.659 (100)	10.282 (100)	11.818 (100)	50.947 (100)

Os motivos de descarte apresentaram grande diversidade. As 16 principais causas totalizaram 76,8% das bolsas desprezadas (Tabela 3), com destaque para volume fora dos parâmetros (7.599 unidades, 14,9%) e estoque excedente (7.454, 14,6%). Essas variáveis foram classificadas em potencialmente evitáveis (11 categorias) e não evitáveis (cinco categorias), com utilização de metodologia utilizada em estudo prévio (14). O primeiro grupo somou 25.784 unidades (50,6%), e a discrepância de volume foi a mais expressiva entre elas.

**Tabela 3:** Principais causas de descarte das bolsas de sangue total ou de hemocomponentes. Hemocentro da Secretaria Estadual de Saúde do Rio Grande do Sul, 2018-2022 (n=50.947)

Natureza	Causas de descarte	Hemocomponente ou sangue total					Total
		Concentrado de hemácias (n)	Concentrado de plaquetas (n)	Crioprecipitado (n)	Plasma fresco congelado (n)	Sangue total (n)	n (%)
Evitáveis	Volume fora dos parâmetros	5	12	3	7.568	11	7.599 (14,9)
	Fora de validade	732	4.327	24	48	-	5.131 (10,1)
	Contaminação por hemácia	-	1.960	-	2.790	-	4.750 (9,3)
	Sorologia reagente	2.652	809	-	478	9	3.948 (7,7)
	Intercorrência no fracionamento	25	376	-	935	13	1.349 (2,6)
	Lipemia	-	159	-	734	43	936 (1,8)
	Presença de grumos	-	882	-	-	-	882 (1,7)
	Doador com conduta de risco	19	-	-	-	559	578 (1,1)
	Sistema aberto	88	38	28	277	66	497 (1,0)
	Ausência de “swirling”	-	101	-	-	-	101 (0,2)
	Armazenamento inadequado	3	3	-	7	-	13 (0,0)
Não evitáveis	Estoque excedente	1	9	-	7.444	-	7.454 (14,6)
	Coloração anormal	-	228	-	8.070	-	8.298 (16,3)
	Utilizada no controle de qualidade	950	703	202	743	-	2.598 (5,1)
	Volume insuficiente de sangue total	-	-	-	-	1.995	1.995 (3,9)
	Voto de autoexclusão	217	76	-	72	256	621 (1,2)
	Subtotal	4.687	9.671	254	21.598	2.941	39.151 (76,8)
	Outras causas	794	312	4	10.420	266	11.796 (23,2)
	Total	5.481	9.983	258	32.018	3.207	50.947 (100)

Para obtenção dos resultados de hemocomponentes aproveitados pelo Hemocentro da Secretaria Estadual de Saúde do Rio Grande do Sul no período (Tabela 4), foram calculados os valores com a utilização dos números de produção com subtração dos descartes. Os componentes com maior aproveitamento foram o concentrado de hemácias, que obteve seu melhor percentual de aproveitamento em 2022 (94,4%), e o crioprecipitado, que representou 93,7% em 2021. Já o plasma fresco congelado obteve os menores rendimentos, com variação entre 17,8% em 2018 e 32,2% em 2019. 

**Tabela 4:** Frequência absoluta (n) e percentual (%) de aproveitamento por hemocomponente. Hemocentro da Secretaria Estadual de Saúde do Rio Grande do Sul, 2018-2022 (n=118.590)

Hemocomponente	Anos				
	2018	2019	2020	2021	2022
	n (%)	n (%)	n (%)	n (%)	n (%)
Concentrado de hemácias	17.300 (93,5)	15.484 (92,5)	14.555 (93,2)	15.432 (94,1)	16.782 (94,4)
Concentrado de plaquetas	2.810 (65,0)	4.166 (74,6)	5.175 (71,8)	5.656 (70,6)	6.889 (72,2)
Crioprecipitado	364 (91,9)	406 (81,4)	218 (80,7)	449 (93,7)	411 (89,0)
Plasma fresco congelado	1.550 (17,8)	2.437 (32,2)	2.616 (30,4)	2.866 (31,4)	3.024 (28,8)

 O impacto financeiro gerado pelos descartes que poderiam ser evitados totalizou R$ 3.508.555, o que equivaleu a US$ 680.506 ou Int$ 1.552.458 (Tabela 5). Em termos anuais, representou R$ 701.711. Foi possível visualizar que o hemocomponente que gerou a maior despesa foi o plasma fresco congelado (45,7%) e a menor, o crioprecipitado (0,2%).

**Tabela 5:** Impacto financeiro gerado pelos descartes potencialmente evitáveis. Hemocentro da Secretaria Estadual de Saúde do Rio Grande do Sul, 2018-2022 (n= 25.784)

Hemocomponente ou sangue total	Valor de referência (R$)	Quantidade descartada	Total		
			R$	US$	Int$
Concentrado de hemácias	150	3.524	528.600	102.525	233.894
Concentrado de plaquetas	135	8.667	1.170.045	226.938	517.719
Crioprecipitado	100	55	5.500	1.067	2.434
Plasma fresco congelado	125	12.837	1.604.625	311.227	710.011
Sangue total	285	701	199.785	38.750	88.400
Total		25.784	3.508.555	680.506	1.552.458

## Discussão

Em 2022, o Rio Grande do Sul teve uma produção total de 103 mil bolsas de sangue (16), das quais 49.321 foram oriundas da capital, Porto Alegre. No mesmo ano, o Hemocentro da Secretaria Estadual de Saúde do Rio Grande do Sul foi responsável por 37,8% da produção do estado e por 78,9% da do município. 

Ainda em 2022, a partir da seleção de hemocentros coordenadores, as unidades federativas que mais produziram bolsas de sangue foram os estados do Rio de Janeiro (n=194.240) e do Paraná (n=447.769) (16). O hemocentro do Rio Grande do Sul apresentou quantidades consideravelmente inferiores.

Estudos mostram que a realização de campanhas, a utilização de cartilhas informativas sobre o processo de doação de sangue, o envio de cartas e de e-mails e a realização de ligações telefônicas auxiliam no aumento da captação de doadores pela sensibilização (17-19).

Quando os anos foram comparados, observou-se que apenas em 2019 houve uma queda em relação às doações do ano anterior. Em 2020, mesmo tendo havido elevação nas doações em relação ao ano que imediatamente o antecedeu, ela ainda ficou abaixo da produção de 2018. Dados mostram que nos anos citados houve uma redução de 10% nos registros de doação de sangue devido ao contexto da pandemia de covid-19 (20). Outro estudo registrou queda de 38% nessa mesma época (21). No Hemocentro da Secretaria Estadual de Saúde do Rio Grande do Sul, a redução não chegou a ser tão expressiva quanto os números obtidos nesses estudos. 

Foi descrito, em relação aos hemocomponentes, que o perfil de componentes sanguíneos necessários para o gerenciamento de estoque coerente e que atenda a demanda solicitada pelas agências transfusionais é de 43% de concentrado de hemácias e 32% de concentrado de plaquetas (22). A produção do hemocentro aproximou-se desse esperado para concentrado de hemácias, uma vez que a média do quinquênio foi de 50,1%. Mas situou-se bem abaixo quanto ao concentrado de plaquetas, que apresentou média de 20,5 %. A pesquisa citada não relatou o perfil para os hemocomponentes crioprecipitado e plasma fresco congelado. 

O hemocomponente mais produzido pelo Hemocentro da Secretaria Estadual de Saúde do Rio Grande do Sul foi o concentrado de hemácias em todos os anos. O dado é compatível com a literatura citada, pois esse é o hemocomponente com maior distribuição. São necessários altos estoques para manter o abastecimento viável.

A partir da avaliação dos boletins anuais de produção hemoterápica da Anvisa (23-25), foi perceptível que as médias de aproveitamento brasileiras e da região Sul do país estiveram bem próximas nos cinco anos avaliados (2018-2022), assim como os valores apresentados pelo hemocentro estudado. São ainda superiores a todos os percentuais nacionais, quando comparado toda a região Sul, e sobrepõem-se no aproveitamento do concentrado de plaquetas e do concentrado de hemácias.

Dos hemocomponentes analisados, o plasma fresco congelado é o que possui menor aproveitamento, paradoxalmente por possuir produção elevada. Pela sua possibilidade de armazenamento por longo período (12 ou 24 meses), apresenta alto percentual de descarte por estoque excedente e pela falta de espaço para armazenamento. Uma alternativa para redução desse descarte é o encaminhamento para a produção de hemoderivados, o qual pode ser realizado por serviços de hemoterapia qualificados (22).

Em 2023, o Hemocentro da Secretaria Estadual de Saúde do Rio Grande do Sul passou a fornecer o plasma excedente para a Empresa Brasileira de Hemoderivados e Biotecnologia (Hemobrás), empresa pública do Ministério da Saúde responsável pela produção de medicamentos hemoderivados no Brasil e pelo fornecimento aos pacientes do SUS. O plasma passou, então, a ter melhor aproveitamento dos hemocomponentes dentro do sistema público de saúde, sem nenhum tipo de comercialização (26).

A maioria dos hemocentros nacionais não faz aproveitamento do sangue total, pois acreditam ser mais eficaz a transfusão de componentes sanguíneos específicos (27). Foi sugerido, contudo, que a transfusão maciça tem se mostrado bastante eficaz, por oferecer uma reposição sanguínea mais completa e abrangente, desde que implementada na prática com os protocolos corretos e profissionais treinados (28).

Em 2018, um relatório com dados gerais nacionais demonstrou que foram produzidas 7.938.696 unidades de hemocomponentes, mas, destas, 33,26% foram descartadas (23). Ao analisar o mesmo ano, o Hemocentro da Secretaria Estadual de Saúde do Rio Grande do Sul apresentou 32,6% de bolsas descartadas, próximo da média nacional. Melhor que o quinquênio anterior, de 2013 a 2017, que apresentou variação de 31% e de 38% nos números de bolsas desprezadas.

Após a separação por motivos de descarte, dos 11 classificados como potencialmente evitáveis, pôde-se atribuir sete (armazenamento inadequado; ausência de swirling; contaminação por hemácia; intercorrência no fracionamento; presença de grumos; sistema aberto; volume fora dos parâmetros) a fragilidades no processo de fracionamento (centrifugação), de controle de temperatura e de armazenamento (2). Esses fatores poderiam ser minimizados com educação permanente para qualificação dos profissionais que executam as funções de fracionamento dos hemocomponentes (29).

A classificação do volume fora dos parâmetros como causa evitável é relativamente controversa, já que ela pode ser causada pela centrifugação incorreta, além de poder depender de parâmetros da amostra. Estudiosos classificaram como evitável considerando que o segundo caso pode ser minimizado com hidratação, boa alimentação e repouso do doador anteriormente à doação (14). 

A situação é idêntica em relação à lipemia, caracterizada pelo aspecto leitoso da amostra, condição que pode ser reduzida com alimentação adequada do doador previamente à coleta do sangue (14). Embora a lipemia não afete diretamente os componentes sanguíneos transfundidos (30), seu descarte ainda é preconizado, pois amostras que contenham essa característica em excesso podem sofrer interferências nos testes laboratoriais sorológicos.

Um estudo descreveu os principais motivos de inaptidão de candidatos à doação de sangue em um hemocentro brasileiro e constatou, em primeiro lugar, o risco para transmissão de doença (36,6%) (13). Dentre outros motivos, descartes devido a doadores com conduta de risco e com sorologia reagente enquadraram-se nessas causas, o que poderia ter sido evitado com disseminação de informações sobre testagem prévia e conscientização (5).

O Hemocentro da Secretaria Estadual de Saúde do Rio Grande do Sul tem uma elevada produção, por isso, deve atender a disposição legislativa que exige que em 1% da produção sejam realizados testes para controle de qualidade (8). Nos resultados deste estudo, observou-se que os descartes para execução do controle de qualidade foram superiores ao exigido (5,1%), o que denotou preocupação elevada em manter o padrão de qualidade, embora signifique um quantitativo alto de bolsas de sangue não utilizadas em transfusões.

Quanto ao descarte de amostras fora da validade, que significa o não aproveitamento de hemocomponentes por vencimento do prazo de utilização, costuma ser elevado, principalmente pelo concentrado de plaquetas, que tem curta viabilidade terapêutica (cinco dias) e alta produção (15). Salienta-se a verificação da necessidade dos serviços para equalizar os níveis de estoque com a demanda real, o que balanceia os períodos do ano com menor rotatividade (22).

Ao se comparar os resultados encontrados neste estudo com outros (15,22), observou-se que os valores e os motivos de descarte foram semelhantes. O maior encontrado foi o de componentes plasmáticos, de 60% a 80%, devido ao excesso de produção, seguido do de crioprecipitado, com 18% a 31% de descarte, devido ao prazo de validade curto (23), e, por fim, o de concentrado de hemácias, com 6% a 12% de descarte.

O impacto financeiro gerado para o hemocentro é estimado com base em gastos de tempo e de material para coleta e para testagem das bolsas que são descartadas (15).O fornecimento desses hemocomponentes não acontece, e o recurso que seria ressarcido, conforme previsto em portaria (12), acaba não se efetivando.

Outro fator importante para ser considerado é a geração de resíduos, que além de serem prejudiciais ao meio ambiente e à saúde coletiva (14), também geram custos ao hemocentro, já que são fornecidos por empresas especializadas em resíduos biológicos (27).

Segundo os resultados desta pesquisa, no período 2018-2022, o valor que o Hemocentro da Secretaria Estadual de Saúde do Rio Grande do Sul deixou de receber por descartes que poderiam ser evitados foi de cerca de R$ 3,5 milhões, aproximadamente R$ 702 mil por ano. Excluiu-se dessa conta os custos citados anteriormente, que representam desperdício de recursos financeiros.

Apesar da elevada produção e do alto percentual de aproveitamento do Hemocentro da Secretaria Estadual de Saúde do Rio Grande do Sul, notou-se que os descartes representaram desperdícios financeiros expressivos. Medidas educativas, como campanhas informativas e capacitações profissionais, são recomendadas para reduzir perdas e melhorar a disponibilidade de hemocomponentes à população. É preciso ressaltar que os descartes evitáveis também afetam os estoques, o que leva a redução das bolsas que poderiam ser ofertadas aos serviços de saúde, prejudicando a população que seria beneficiada com as doações. 

## Data Availability

O banco de dados e as referências utilizadas na análise da pesquisa podem ser encontrados em https://osf.io/w58hq.
